# Investigating the Association of Quantitative Gait Stability Metrics With User Perception of Gait Interruption Due to Control Faults During Human-Prosthesis Interaction

**DOI:** 10.1109/TNSRE.2023.3328877

**Published:** 2023-12-04

**Authors:** Amirreza Naseri, I-Chieh Lee, He Huang, Ming Liu

**Affiliations:** University of North Carolina at Chapel Hill and North Carolina State University Joint Department of Biomedical Engineering, North Carolina State University, Raleigh, NC 27695 USA

**Keywords:** Gait stability measurements, internal disturbance in robotic prosthesis, powered knee prosthesis, individuals with transfemoral amputation, gait stability perception

## Abstract

This study aims to compare the association of different gait stability metrics with the prosthesis users’ perception of their own gait stability. Lack of perceived confidence on the device functionality can influence the gait pattern, level of daily activities, and overall quality of life for individuals with lower limb motor deficits. However, the perception of gait stability is subjective and difficult to acquire online. The quantitative gait stability metrics can be objectively measured and monitored using wearable sensors; however, objective measurements of gait stability associated with human’s perception of their own gait stability has rarely been reported. By identifying quantitative measurements that associate with users’ perceptions, we can gain a more accurate and comprehensive understanding of an individual’s perceived functional outcomes of assistive devices such as prostheses. To achieve our research goal, experiments were conducted to artificially apply internal disturbances in the powered prosthesis while the prosthetic users performed level ground walking. We monitored and compared multiple gait stability metrics and a local measurement to the users’ reported perception of their own gait stability. The results showed that the center of pressure progression in the sagittal plane and knee momentum (i.e., residual thigh and prosthesis shank angular momentum about prosthetic knee joint) can potentially estimate the users’ perceptions of gait stability when experiencing disturbances. The findings of this study can help improve the development and evaluation of gait stability control algorithms in robotic prosthetic devices.

## I. INTRODUCTION

The field of Human-Machine Interface (HMI) aims to develop technologies for human-machine interaction. One notable application is assistive devices for individuals with walking-related disabilities [1], [2]. Robotic exoskeletons and prostheses are compelling examples that enhance mobility and independence [3], [4]. These devices use various control methods to align with user intentions and daily activities [5], [6], [7], [8]. However, they encounter challenges like the risk of falls and injuries due to internal faults [9], [10], arising from control and sensor failures in unpredictable environments, which can disrupt walking gaits [11], [12]. Hence, it is essential to evaluate robotic assistive devices’ performance and their impact on the user’s dynamic gait stability in real time.

Subjective perception of the robotic assistive device’s functionality and its impact on walking gait stability directly affect users’ psychosocial conditions, such as fear of falling and reduced physical activity [13], [14], [15]. These subjective perceptions are widely applied to guide prosthesis tuning in clinics and even used to directly tune control parameters of robotic assistive devices [8], [16], [17]. Also, this subjective perception can be very sensitive to the gait stability interruption due to the joint-level disturbances compared to objective measurements [18]. Nonetheless, these subjective measurements have large variations and are difficult to access in real time. This poses a challenge in using them as the primary feedback mechanism for the control of robotic assistive devices.

On the other hand, dynamic gait stability can be quantified objectively using motion capture systems. Gait stability in the context of human locomotion refers to the capacity to preserve or reestablish an upright posture without necessitating changes to the current base of support (BOS), when confronted with internal or external perturbations [19]. There are many objective metrics proposed for quantifying gait stability in walking including: 1) spatiotemporal parameters (step length, step width, step time) [20], [21], 2) stability parameters (i.e., full-body angular momentum about the center of mass, the margin of stability, and inclination angle) [22], [23], [24], [25], 3) Lyapunov exponent [26], 4) maximum floquet multipliers [26], 5) detrended fluctuation analysis [27], 6) postural control parameters (i.e., the center of pressure [28] and center of mass displacement [29]), and approximate entropy [30]. In general, there is no “gold standard” among these metrics for gait stability quantification. The use of the metrics depends on the specific applications.

Focusing on robotic assistive devices, objective measures have often been used as a feedback signal in control or to evaluate the device’s effectiveness. For instance, the margin of stability was used to evaluate the effectiveness of a powered hip exoskeleton in recovering balance after introducing disturbance as forward/backward pull [31]. The margin of stability was also investigated for the active pelvis orthosis after applying the foot slippage during treadmill walking [32]. Spatiotemporal parameters, the margin of stability, and center of mass trajectory were evaluated in case of disturbance as forward push while using powered ankle exoskeleton [33].

These objective measures do not incorporate subjective feedback from the user. Such misalignment between the objective gait stability metrics and the subjective perception of gait stability may make it harder to improve users’ confidence by mitigating disturbance through intervention (that relies on objective measurements). It remains an open question which objective stability metrics are more closely associated with human perception of gait stability during walking. To our knowledge, there have been very limited studies investigating the correspondence between objective metrics and users’ subjective experience of dynamic gait stability.

Consequently, this study endeavors to bridge this knowledge gap by embarking on a systematic exploration of the association between various gait stability metrics and the subjective assessments of gait stability. We chose abnormal control parameters that can cause internal errors on robotic prostheses as the source of disturbance because similar errors can be generated if the prosthetic devices cannot interpret users’ intentions reliably (a challenging issue in the control of the robotic prostheses) [9], [34], [35].

Here, we focused on gait stability metrics, which can be measured through wearable sensing systems in real time. Because stability metrics, such as Lyapunov exponent, maximum floquet multipliers, detrended fluctuation analysis, and approximate entropy, rely on relatively large amounts of data to get an accurate estimation [36], we did not include them in this study. Although we noticed that local kinematics, such as thigh motion, can be affected by the disturbance too, we adopted a more general local kinematic variable, knee joint momentum, which reflects both the prosthetic thigh and shank motion, instead of kinematics of single joint or segment.

The major contribution of this study includes (1) investigating the effects of internal control disturbance on the various measures of gait stability and prosthesis local parameters and (2) identifying metrics that are associated with the prosthesis users’ gait stability perception while interacting with robotic prostheses operating under normal and disturbed strides. Identifying gait stability metrics linked to user perception may enhance our understanding of how individuals perceive the functional outcomes of robotic assistive devices, such as prostheses. The identified metrics can be exploited for monitoring the influence of internal control disturbance on prosthesis functionality. Also, the results can be utilized to guide fault-tolerance strategies (intervention control schemes) that can be implemented in robotic prosthetic legs in future studies.

## II. METHODS

### A. Participants

The research project was reviewed and approved by the University of North Carolina at Chapel Hill Institutional Review Board, under protocol number 13-2689, with an approval date of April 8, 2021. Inclusion criteria for participants were as follows: exhibiting a K3 or K4 level of functional classification, indicative of unilateral transfemoral amputation, attaining a minimum age of 18years, free from cognitive impairment and serious illnesses (i.e. stroke, severe heart disease). Seven participants, who had unilateral transfemoral amputations, gave written consent to participate. The demographics of the participants are listed in Table I. The term “users” will refer to individuals with transfemoral amputation who used prostheses in this paper. Before the first day of the experiment, each subject went through five sessions to get familiar with walking using the NREL-A1 [37] prosthesis under the supervision of a certified prosthetist. These sessions entailed achieving speeds higher than 0.6m/s for overground walking without additional support and assistive devices.

### B. Experimental Setup and Measurements

A robotic transfemoral prosthesis (NREL-A1 [37]), including a powered-actuated knee joint and a passive ankle joint, was used in this study. During the experiments, subjects walked on level-ground while optimizing impedance parameters in each state of a finite state machine (FSM) controller of the prosthesis. The FSM controller, as described in [37] encompasses three states during the stance phase: Initial Double Support (IDS), Single Support (SS), and Terminal Double Support (TDS), as well as two states during the swing phase: Swing Flexion (SWF) and Swing Extension (SWE). Additionally, parameters specific to ramp walking were recorded for reference. The control parameters for ramp walking could introduce disturbances if applied during level ground walking.

The prosthesis users were equipped with an Inertia Measurement System (IMU, Xsens North America Inc, El Segundo, CA, US) for capturing full-body kinematics besides an in-shoe pressure distribution measurement system (PEDAR, Novel, Germany) for measuring ground reaction force and center of pressure. In addition, a potentiometer (RDC503013A, ALPS, Japan) and a load cell (Mini58, ATI, NC, US) were embedded in the NREL-A1 to measure knee angle and dynamical loads/forces on the prosthesis.

### C. Experimental Protocol

There were two sections for the experiments. The first section determines the control (impedance) parameters for each subject, which can be used to generate the disturbance. To create these disturbances, we defined a disturbance vector based on the differences between the impedance control parameters for level ground walking and the ones for ramp walking. We then scaled the disturbance vector and added it to the control parameters of level ground walking to generate the impedance parameters needed to apply the disturbances [35]. The direction and amplitude of the disturbances can be controlled by the scaling factor. The absolute values of the scaling factor were increased gradually to generate disturbances with various severity/intensity.

**Fig. 1.**
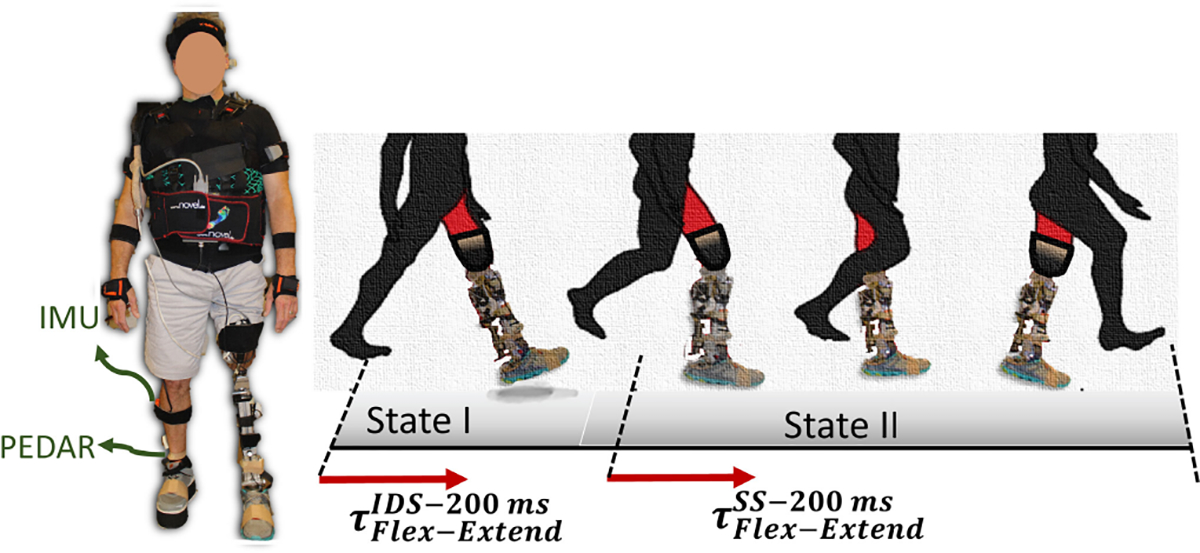
Experiment protocol to analyze the human and prosthesis response to the internal disturbances. Data was collected from the level ground walking experiment of subjects with transfemoral amputation. The disturbances $\left(\tau_{\text{Flex}-\text{Extend}}^{\text{IDS/SS}-200\text{ms}}\right.$) were manually applied for 200 ms in initial double support (IDS) or single support (SS) states of stance phase to generate flexion or extension disturbance torques [35], [40].

The participants were instructed to walk on an 8-meter walkway (7–8 gait cycles) at their own pace. During walking, impedance parameters for applying disturbance were introduced for 200ms randomly in one of the gait cycles (Fig. 1). When participants reached the end of the walkway, they provided feedback to assess the effect of the disturbance on their walking stability. The participants were instructed to evaluate the level of disturbance as “none” (not detectable machine faults), “small” (detectable but preserves stable gait), “medium” (noticeable but recoverable instability), and “large” (significant instability with difficult recovery). We chose this scaling approach over other subjective self/clinical-report measurements, such as the Activities-specific Balance Confidence (ABC) and Berg scale, as these clinical metrics primarily gauge balance confidence in performing various activities [38], [39].

After observing that a particular impedance parameter could generate the same level of disturbance perception on the users’ gait stability at least twice, we recorded the corresponding impedance values for each disturbance condition. Using this approach, we selected twelve disturbance parameters, representing twelve conditions for each participant: two disturbance types (flexion and extension torque), two disturbance timings (IDS and SS phase), and three intensity levels of disturbances on their gait stability (reported as none/small, medium, and large).

In the data collection section (second section of the experiment), participants followed the same procedure while walking on the 8-meter walkway and reported the intensity level of disturbance on their gait stability when they reached the end of the walkway. During each trial, they walked back and forth three times, with six disturbance conditions (2 types and 3 intensity perceptions) applied in a randomized order. To counterbalance the order of timing conditions, four participants began the experiment with disturbances applied during the IDS phase. Each subject repeated trials 28 times, resulting in approximately 168 disturbances. A very small number of disturbed strides were discarded due to human operational errors (e.g., applying the disturbance twice) or sensor issues (e.g., IMU sensor drop). Disturbances categorized as “medium” and “large” were considered perceivable due to their potential impact on user gait stability, while “none” and “small” were classified as non-perceivable/imperceptible disturbances. Adequate rest periods were incorporated between trials to prevent fatigue. Participants were also equipped with a harness system to ensure safety. Additional details can be found in [35].

The number of reported disturbance level were presented in Table II. Detailed discussions regarding the potential reasons for variations in how each subject reported each intensity level will be provided in Sec. IV. Discussion.

### D. Gait Stability Measurements

After collecting data, we analyzed various gait stability metrics to investigate which get affected by the generated disturbance and how closely they are related to the prosthesis user’s perception of their own walking stability during normal and disturbed walking. Since the disturbances were applied as a flexion/extension type at the knee joint, the focus is to analyze the users’ gait stability in the sagittal plane. Also, we focused on the gait stability metrics that can be continuously monitored using wearable sensors suitable for real time control purposes. Hence, the metrics that were used in this study were divided into four categories based on their underlying mechanisms and theories: 1) spatial parameters including step length and step width; 2) stability parameters including the margin of stability (MoS), the inclination angle, and whole-body angular momentum about the center of mass; and 3) postural control parameters such as the vertical displacement of the center of mass (V-CoM) and the anterior-posterior progression of the center of pressure (A-P CoP) [22], [38]. In addition to these global gait stability metrics, we also analyzed 4) the angular momentum at the knee joint (local measurement). From a mechanical perspective, when the additional disturbance torque is applied to the knee joint, the effects should emerge in the shank and thigh motions (angles, velocities, and accelerations), instantaneously. Knee momentum encapsulates the collective behavior of all the segments connected with the disturbed joint (instead of individual joint kinematics). Therefore, the angular momentum of the adjacent segments at the prosthetic knee joint may be a reliable measurement that can spontaneously represent the impact of the disturbance on that joint.

In the following, we described the underlying mechanism and the measurement procedure of each gait stability metric. Walking without disturbance and while experiencing disturbances called herein normal and disturbed walking conditions, respectively. The comparison of each gait stability metric in disturbed and normal walking conditions is elaborated in sec. II-E. Gait Stability Metric-Perception Association Analysis.

#### 1) Spatial Parameters:

##### a) Step Length and Step Width:

**Fig. 2.**
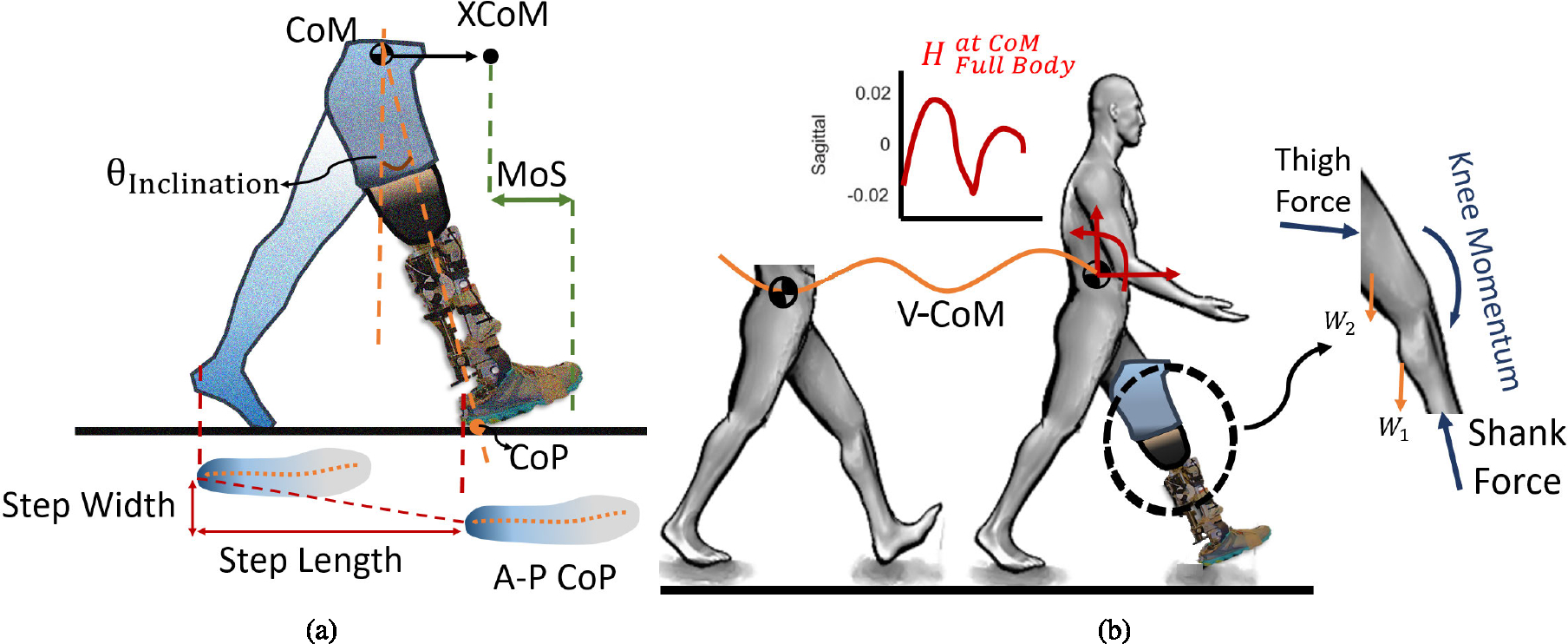
Schematic representation of the metrics used to investigate the effect of the internal disturbances on gait stability. (a) Step Width, Step Length, Margin of Stability (MoS), Inclination Angle ($\Theta_{\text{Inclination}}),$ and Anterior-Posterior (A-P) progression of Center of Pressure (CoP). Step length and step width were measured from the heel strike of the prosthetic foot to the consecutive heel strike of the intact limb. The MoS determined as the distance of the extrapolated center of mass (XCoM) to the toe position of the prosthetic foot that represents the boundary of the base of support. The inclination angle represents the CoM-CoP separation and is measured from the angle in between the line passing from prosthetic foot CoP and CoM and the vertical axis. A-P CoP is defined as the displacement of CoP from heel to toe during the stance phase of the prosthetic foot. (b) Vertical Center of Mass (V-CoM) trajectory, Full-Body Angular Momentum around body CoM, and residual thigh and prosthetic shank momentum around the knee joint (Knee Momentum). The knee and full-body angular momentum were calculated in sagittal planes.

These parameters reflect the individual’s ability to consistently orient their lower extremity in space [41], [42], and they can be further divided based on the direction of control: the automatic passive mechanisms govern the lower extremity placement in the anterior-posterior direction (step length), while active mechanisms control the placement in the mediolateral direction (step width) [38]. Using the insole pressure sensor, the heel contact of each foot was detected and the corresponding step length and step width were determined as shown in Fig. 2. The step length and step width were calculated over two consecutive steps initiated by the prosthetic foot. These measurements were normalized to the user’s leg length.

#### 2) Stability Parameters:

##### a) Margin of Stability:

An extrapolated center of mass (XCoM) which is a parameter that combines both the CoM position and velocity in relation to the base of support can be considered to measure an individual’s stability (Eq. 1) [43], [44].


(1) $$XCoM=CoM+\frac{{vel}_{CoM}}{\sqrt{\frac{g}{l}}}$$

In which ${vel}_{CoM}$ is the CoM velocity in Anetrior-Posterior direction and l is the leg length.

The margin of stability is the maximum deviation of the XCoM from its base of support that can be tolerated without losing balance [23]. The toe trajectory was considered as the edge of the base of support and the corresponding margin of stability was defined as depicted in Fig. 2. The margin of stability was calculated over the stance phase of the prosthetic foot and normalized to the excursion value (min-max difference) of the respective mean of normal profiles.

##### b) Inclination Angle:

The orientation of the line connecting the center of pressure (CoP) and center of mass (CoM) at any given moment can describe the body’s orientation relative to the supporting foot during locomotion. When this line is referenced to a vertical line passing through the CoP, the inclination angle in the sagittal plane can be defined [38]. This angle takes into account both the CoM’s instantaneous height in addition to the horizontal distance between the CoM and CoP [45], [46]. The inclination angle was measured during the stance phase of the prosthetic foot for both normal and disturbed walking conditions and then was normalized to the excursion value (min-max difference) of the respective mean of normal profiles.

##### c) Full-Body Angular Momentum about Body Center of Mass:

The full-body angular momentum about CoM is a measure of the rotational motion of the body around its center of mass. In [22], it was shown that during level-ground and treadmill walking, the full-body angular momentum about CoM remains relatively constant. However, due to environmental perturbation such as uneven terrain, the full-body angular momentum would change as the body adjusts to maintain balance.

To calculate the full-body angular momentum, a 14-segment human model (including head, trunk, lower arms, upper arms, hands, upper legs, lower legs, and feet) was considered. Segmental CoM and full-body CoM kinematics were derived by full-body IMU measurement and plugged into Eq. 2 [47]:

(2) $${\vec{H}}_{Body}^{CoM}=\sum_{i}^{n}\left[I^{i}{\vec{\omega }}^{i}+{\vec{r}}_{CoM}^{i}\times \left(m^{i}\left({\vec{V}}^{i}-{\vec{V}}^{CoM}\right)\right)\right]$$

in which ${\vec{H}}_{Body}^{CoM}$ is full-body angular momentum about the CoM, $I^{i}$ is the $i^{th}$ segment moment of inertia tensor about its center of mass, ${\vec{\omega }}^{i}$ is the $i^{th}$ segment angular velocity vector, ${\vec{r}}_{CoM}^{i}$ is the $i^{th}$ segment center of mass distance to the body CoM, $m^{i}$ is the $i^{th}$ segment mass, and ${\vec{V}}^{i}$, and ${\vec{V}}^{CoM}$ are the $i^{th}$ segment center of mass linear velocity and body CoM linear velocity. All parameters are with respect to the fixed reference frame. The full-body angular momentum about CoM was calculated over the full gait cycle of the prosthetic foot and normalized to the user’s height and weight.

#### 3) Postural Control Parameters:

##### a) Center of Pressure Progression in Anterior-Posterior Direction:

The center of pressure is a point on the ground where the total force from the ground is applied. It provides information about the distribution of forces and the stability of the body during movement. So, it can be used to quantify an individual’s gait stability [48]. The A-P CoP trajectory was measured over the stance phase of the prosthetic foot and normalized to the foot length.

##### b) Center of Mass Displacement in Vertical Direction:

The center of mass is a point that represents the application of the gravitational and internal forces on the body. One important application of the center of mass is maintaining balance in the analyses of locostationary and locomotory behavior during everyday tasks. So, by measuring the vertical displacement of the center of mass during walking, it is possible to analyze how the walking gait stability changes during different types of locomotion [38], [48], [49]. The vertical displacement of CoM was calculated over the full gait cycle of the prosthetic foot and normalized to the user’s height.

#### 4) Prosthetic Thigh and Shank Angular Momentum About Prosthetic Knee Joint (Knee Momentum):

In the context of gait, it is possible to determine how each segment is reacting to the disturbances by examining the angular momentum of the body’s segments. In our study, different types of disturbance torques in the sagittal plane were applied to the knee joint. So, it would be reasonable to investigate the angular momentum of adjacent body segments around the knee joint. These local segments can provide information on instantaneous responses to the applied internal disturbance. To calculate the momentum at the knee, the angular momentum of the residual thigh and the prosthetic shank was determined about the knee joint using Eq. 3 (adopted from [50]):

(3) $$\begin{array}{l} {\vec{H}}_{/Knee}^{Shank\;and\;Thigh}\\ \;=[I^{Shank}{\vec{\omega }}^{Shank}+{\vec{r}}_{Knee}^{Shank}\times m^{Shank}{\vec{V}}^{Shank}]\\ \;+[I^{Thigh}{\vec{\omega }}^{Thigh}+{\vec{r}}_{Knee}^{Thigh}\times m^{Thigh}{\vec{V}}^{Thigh}] \end{array}$$

in which ${\vec{H}}_{/Knee}^{Shank\;and\;Thigh}$ is the shank and thigh angular momentum about the knee joint, I and m are the moment of inertia tensor and mass of each segment, respectively. $\vec{\omega }$ and $\vec{V}$ represent the segment center of mass angular and linear velocity, accordingly. The knee joint angular momentum was determined over the full gait cycle of the prosthetic foot and normalized to the user’s height and weight.

### E. Gait Stability Metric-Perception Association Analysis

#### 1) Data Pre-Processing:

In order to define the association between the quantitative metrics and gait stability perception, we performed the following data preprocessing: the data collected from sensors embedded in the NREL-A1 prosthesis and an insole pressure sensor (PEDAR) was logged at 100Hz, while IMU data was logged at 60Hz. To achieve synchronization, the prosthetic leg controller generated a synchronization signal that triggered the recording of data by the IMU and PEDAR systems. Simultaneously, this synchronization signal was also recorded by the prosthetic leg. A second-order Butterworth filter with a cut-off frequency of 7Hz was applied to smooth all measurements. Subsequently, we computed gait stability metrics and divided them into strides based on the 100% of the gait cycle.

To evaluate the effect of disturbance on each gait stability metric, we compared the patterns/trajectories of these metrics in disturbed conditions to the average pattern of normal strides (360–380 gait cycles). The average pattern of normal strides is derived from the mean of all gait cycles, excluding perturbed stride and a subsequent stride. This average pattern served as the baseline for comparing gait stability metrics during disturbed gaits. The deviation of each gait stability metric under disturbed conditions from this baseline (average of the normal pattern) was quantified as follows:

- We trimmed the data of disturbed strides from the time of disturbance application to the end of the prosthetic foot’s stance phase.
- For each disturbed condition, we scaled the average of normal strides to match the size and timing of the disturbed stride.
- To ensure uniformity and comparability across gait parameters, the root mean squared error (RMSE) was used to measure 1) the variation of normal strides relative to the average of normal strides and 2) the difference between disturbed strides and the average of normal strides.
- We normalized the RMSE to the excursion value (min-max difference) of the average of normal strides, resulting in normalized RMSE (N-RMSE).

#### 2) Statistical Analysis:

As mentioned, there were twelve disturbance conditions in this study; two disturbance types (flexion and extension), two disturbance timing (IDS and SS), and three perception levels (“none/small”, “medium”, and “large”). Due to the fact that participants can employ different walking/recovery strategies to compensate for internal prosthetic disturbances (details will be discussed in Sec. IV. Discussion), one condition may be sensitive to one participant but not to others. Consequently, individual analyses were conducted in this study.

Given the design of the experiment with disturbances applied at different timings and types, it was unknown if all the disturbance conditions have a distinct effect on the participant and whether each condition needs to be treated differently. Therefore, statistical parametric mapping (SPM) analysis was conducted to answer this question [51]. The perception levels were determined by the report of participants at the first visit, so the intensity effect is already confirmed.

SPM is a statistical approach that allows the identification of regions of parameter space (for continuous data) that are statistically significant [52]. Using SPM, we evaluated the effect of timing of disturbance application (IDS or SS) and type of disturbance (flexion or extension) conditions on the gait stability metrics, such as comparing the effect of the same intensity/severity and timing but different types of disturbance or the effect of the same types and severity but different disturbance timing. In SPM, for each paired-wised comparison between patterns of disturbed strides having similar conditions except timing or except type, we focused on the time window from the initiation of disturbance at IDS to the end of the stance phase. In cases where the disturbance occurred during SS for both disturbed conditions, we considered the time window from the start of the disturbance in SS to the end of the stance phase. SPM analysis assessed whether there were significant differences in the gait patterns during the specified time window. A significance level of 0.05, was used to determine whether certain portions or intervals of time exhibited statistically significant differences between the two compared patterns. At the end, conditions that showed no significant difference were then merged, resulting in a more general disturbance case.

To find the association of the objective measurements to subjective reports of gait stability, first, a linear Kendall correlation was performed. With this analysis, we assessed the association between N-RSME of gait stability metrics (continuous data) and users’ perception of disturbance intensity levels on their gait stability (ordinal outcomes) [53]. Kendall correlation coefficient was used as it provides a robust measure of correlation, suitable for the goal of this study.

Second, to assess how changes in the N-RMSE of selected gait stability metrics relate to the probability of perceivable disturbances (disturbance reported as “medium” or “large”), the generalized linear model (GLM) using binomial logistic regression [54] was applied. The percentage is complementary to the percentage where the user may report the disturbance as non-perceivable (“none”/“small”). The GLM equation for modeling the probability of perceivable disturbances with the gait stability metrics for the $i^{th}$ participant during disturbance condition $j^{th}$ is:

(4) $$P(perceivable\;disturbance{)}_{ij}=\frac{e^{\beta_{0ij}+\beta_{1ij}x_{ij}+\beta_{2ij}x_{ij}^{2}}}{1\;+\;e^{\beta_{0ij}+\beta_{1ij}x_{ij}+\beta_{2ij}x_{ij}^{2}}}$$

where $x_{ij}$ is the calculated N-RMSE of a gait stability metric for the $i^{th}$ subject with $j^{th}$ disturbance condition. The continuous probability (P) for the perceivable disturbance generated in the log-odds scale. We defined the explanatory variables $\left(x_{ij}\right)$ as a sum of the given N-RMSE of degree 0, 1, and quadratic term. This choice was based on the anticipation that the probability would exhibit an initial increase with the magnitude of N-RMSE and eventually reach a saturation point. For assessing the goodness of fit of the model, we employed pseudo-R squared [55]. Pseudo-R squared provides insights into the model’s fit by quantifying the proportion of variance explained by the model. In our analysis, higher pseudo-R squared values indicate a better fit, signifying that the model captures a larger portion of the data’s variance.

For each subject, we determined the Kendall correlation coefficient and the pseudo-R squared for the metrics; the metrics showing a higher correlation coefficient compared to other metrics and pseudo-R squared higher than 0.2 [56] being consistently identified among the majority of subjects can be regarded as better associated with users’ perceptions of their gait stability.

## III. RESULTS

### A. Effects of Disturbances on Gait Stability Metrics

**Fig. 3.**
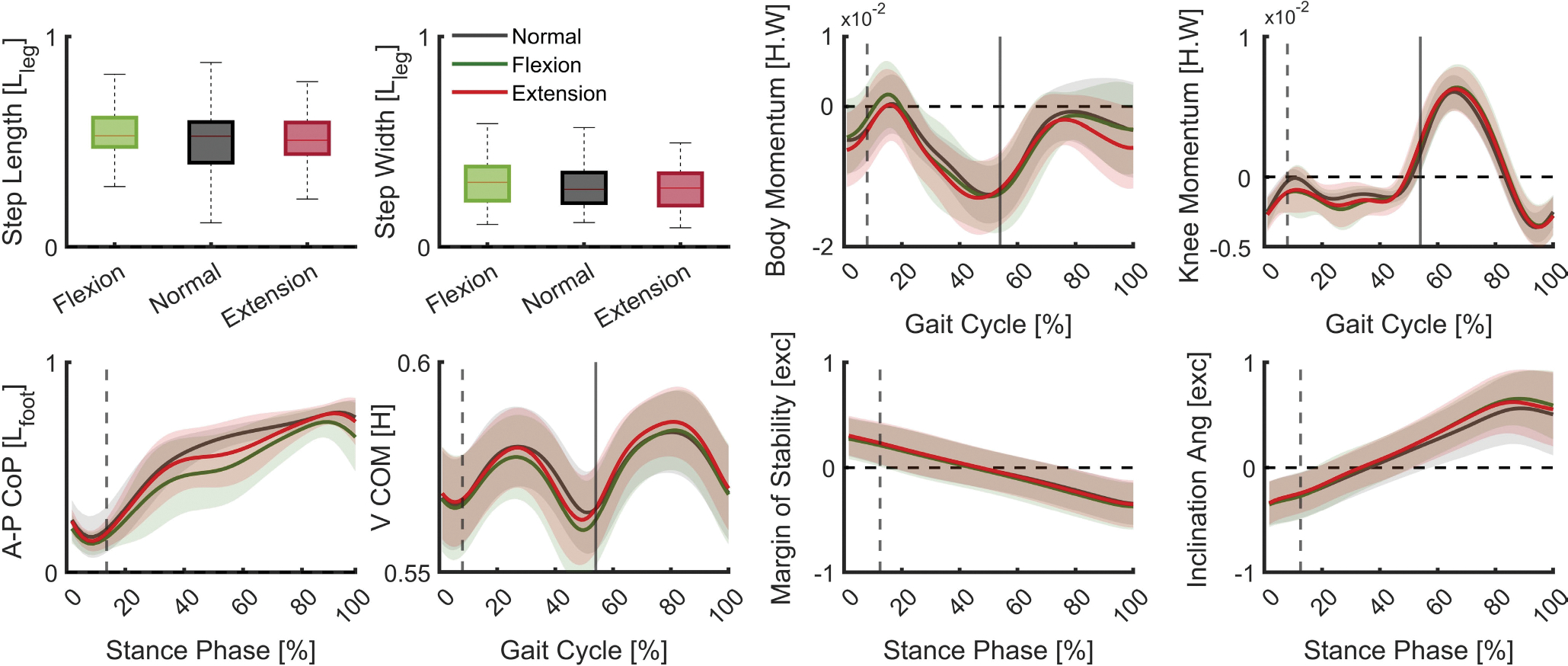
Gait stability patterns in normal walking (black) and disturbed walking conditions when the disturbance type is the extension (red) and the flexion (green). The gray dashed line is the time the disturbance applied at IDS. The solid line represents the end of the stance phase. The top row indicates the prosthetic leg step length and step width, full-body angular momentum about CoM, and thigh and shank of prosthetic side momentum around the prosthetic knee joint (knee momentum). The step length and step width were measured between two consecutive heel strikes while full-body and knee angular momentum were measured during one gait cycle. The bottom row presents the center of pressure progression of the prosthetic foot in the sagittal plane (Anterior-Posterior direction, A-P CoP), vertical displacement of the body center of mass (V-CoM), the margin of stability (MoS), and inclination angle. The A-P CoP, inclination angle, and MoS were measured during the stance phase of walking while V-CoM was determined during one gait cycle. The profiles are based on the data of all 7 subjects. [ ] indicates normalization. exc is the abbreviation of excursion.

Figure 3 provides kinematic magnitude/trajectories of the selected gait stability metrics over one gait cycle or stance phase of the prosthetic foot. The profiles include the mean and standard deviation of the normal and the perceivable disturbed strides under different disturbance types.

#### 1) Spatial Parameters:

The average normalized step length during normal walking was found to be 0.59m, while it was 0.60m for flexion disturbances and 0.57m for extension disturbance torques (Fig 3). The step length during normal walking had a significant overlap with the step length during disturbed walking. The step width also displayed a similar trend. During normal walking, the normalized average step width was 0.27m while for flexion and extension disturbances was 0.31m and 0.25m, respectively.

#### 2) Stability Parameters:

##### a) Margin of Stability:

The profiles start from a positive value where the toe is in front of the XCoM and progressively decrease as the toe leaves the ground behind the XCoM. As indicated in Fig. 3, the variability of the margin of stability for the normal strides covered the profiles of the disturbed strides during the stance phase.

##### b) Inclination Angle:

The patterns show a gradual increase until the heel-off event and a decrease in angle as the foot leaves the ground. There is a deviation between the normal profiles and the patterns corresponding to the flexion and extension disturbances during the late stance phase. However, it is within the variation of normal profiles. This deviation in inclination angle may be due to the disturbance propagation to the body and its impact on posture as a result of the applied disturbance.

##### c) Full Body Angular Momentum about Body Center of Mass:

The patterns of full-body angular momentum for normal walking closely resemble those observed in individuals without walking impairments [22]. The direction of body orientation, as represented by the positive and negative slope of angular momentum profiles, is depicted in Fig 3. While there is a deviation between the normal walking pattern and disturbed strides, the variation of normal patterns is sufficient to encompass these deviations. The largest deviation is observed during the late swing phase which can represent the time latency to materialize the propagation of disturbance to the whole body posture.

#### 3) Postural Control Parameters:

The comparison of gait stability metrics as shown in Fig. 3 reveals that both the A-P CoP and V-CoM metrics exhibit the most deviation from the normal profiles after the disturbance is introduced. The average disturbed patterns for both V-CoM and A-P CoP due to flexion and extension disturbances are lower than those of the normal patterns during the stance phase. For A-P CoP, there is a smooth progression of the CoP from heel to toe; however, this progression stagnates for the disturbed strides as there is a braking or backward movement of the prosthetic A-P CoP. Similarly, for the V-CoM, both disturbance types cause the user to lower their body (lower V-CoM compared to normal walking).

#### 4) Prosthetic Thigh and Shank Angular Momentum About Prosthetic Knee Joint (Knee Momentum):

The sign of the slope of the knee momentum trajectory reflects the direction of rotation, whether it is flexion or extension. The peak of angular momentum occurs at the toe-off event, which is consistent across all gait patterns. The deviation of the angular momentum patterns between flexion and extension disturbances and the normal strides is most pronounced immediately after the disturbances were applied.

### B. Association of Gait Stability Metrics to User Perception

**Fig. 4.**
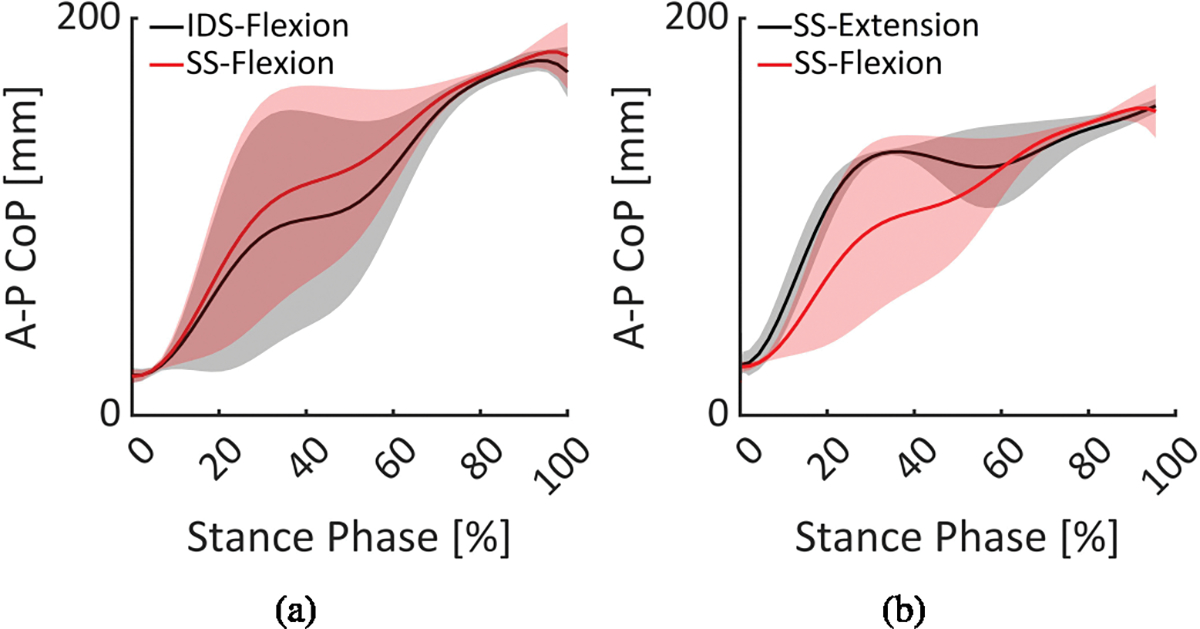
Statistical Parameter Mapping (SPM) for pair-wise comparison between A-P CoP patterns. The comparison was performed during level ground walking as the disturbance was applied with different types (flexion and extension) and timing (IDS and SS). The representative example of perceivable a) flexion-type disturbance applied at the IDS (black) and SS (red), and b) extension (black) and flexion (red) disturbances were applied at the SS of the stance phase is shown.

The SPM analysis was performed and the results revealed that the disturbance with different timing (IDS or SS) can be combined (Fig. 4). For instance, when both extension disturbances were applied at the IDS and SS, there was no significant difference in the patterns of metrics. The same was true for flexion disturbances. However, when flexion disturbances were applied at the IDS and compared to the pattern in which extension disturbances were applied at the IDS, a significant difference was observed between the patterns. Similar results were obtained when different disturbance types were applied at the SS. The data were then categorized based on the disturbance type (combining data of different timing).

Consequently, Kendall correlation analysis and binomial logistic regression models were performed for flexion and extension type disturbances separately. The results of the Kendall correlation reveal that for the flexion type disturbance, the subjective perception showed an association with the:

- knee momentum of TF01, TF02, TF06, TF07,
- A-P CoP of TF01, TF02, TF03, TF07,
- margin of stability of TF05, TF07,
- V-CoM of TF07, • full-body momentum TF01, TF07, and
- inclination angle of TF01, TF07.

where the correlations reach significance. Also, for the extension type disturbances, the results showed a significant correlation for:

- knee momentum of TF01, TF03, TF04, TF05,
- A-P CoP of TF01, TF02, TF06,
- inclination angle of TF06,
- V-CoM of TF03, TF04, and
- full-body momentum of TF04.

**Fig. 5.**
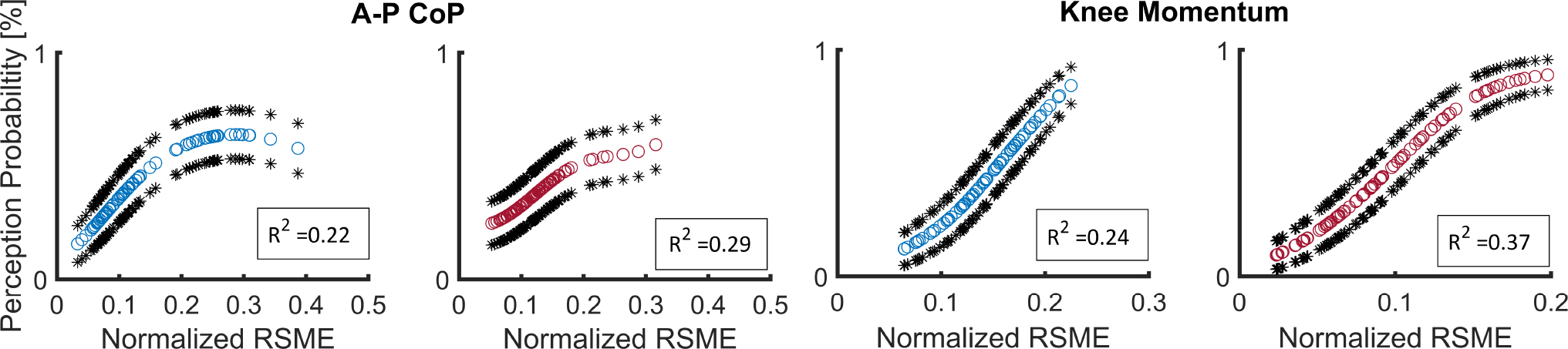
Binomial logistic regression model to predict the probability of the perceivable disturbances when the probability exponentially fits the N-RMSE of A-P CoP and knee momentum for TF01. The prediction results for flexion and extension disturbances are shown in blue and red colors correspondingly (with a 95 confidence interval represented by black stars). The N-RMSE is the root mean square error normalized to the respective excursion (max − min) value of the average of the normal pattern.

In addition, Fig. 5 illustrates representative results of the binomial logistic regression model of TF01 showing the trend of change between the probability of reporting perceivable disturbance (reporting perception of medium or large) for knee momentum and A-P CoP. Among all the calculated metrics, only knee momentum and A-P CoP exhibits higher pseudo-R squared $\left(R_{GLM}^{2}>0.2\right)$ for the majority of the participants (knee momentum for flexion disturbance: TF01, TF02, TF06, and TF07; knee momentum for extension disturbance: TF01, TF03, TF04, and TF05; A-P CoP for flexion disturbance: TF01, TF02, TF03, and TF07; A-P CoP for extension disturbance: TF01, TF02, and TF06).

Table III and IV in the supplementary material present the correlation coefficients and their corresponding significance levels for each gait stability metric, separately for flexion and extension disturbances, across all subjects. Additionally, Tables V and VI in the supplementary material provide the pseudo-R squared values for each gait stability metric and each subject, categorized by the type of disturbance (flexion or extension).

## IV. DISCUSSION

The study presented in this paper aimed to evaluate the effectiveness of various gait stability metrics associated with prosthetic users’ perception of gait stability in the case of internal control disturbance applied at a powered knee prosthesis. Ensuring that gait stability metrics are associated with the users’ own perception is a crucial aspect of evaluating gait performance in individuals with lower limb amputation. By identifying such metrics, we can gain a more accurate and comprehensive understanding of an individual’s perceived functional outcomes of robotic assistive devices such as prostheses.

In order to define the metrics associated with the users’ perception, we conducted a comparative analysis between gait stability metrics determined for normal strides and those affected by the internal disturbances, during the onset of the disturbance until the end of the prosthetic stance phase. This time window encompasses a 200ms period during which the disturbance was applied and the subsequent voluntary recovery response of the users. The aim of evaluating the comparison for this period of time allows us to identify which metrics are more sensitive to short-duration disturbances and user responses, which can provide insights into the potential targets for real time feedback control and internal disturbance intervention.

The results of our study indicate that the knee momentum and A-P CoP are parameters that were more closely associated with users’ perceptions of their own gait stability during internal disturbances for the majority of subjects. Knee momentum is a newer metric that has not been widely used in gait assessment. However, our study suggests that for a joint-level disturbance, it may be useful to consider a local parameter, proximal to the disturbed joint, in addition to global gait stability metrics.

In our experimental design, we aimed to maintain an equal distribution of disturbance control parameters, resulting in the same number of intensity levels reported by all subjects. However, it became evident that users sometimes reported inconsistent perceptions (Table II) even when exposed to the same control parameters corresponding to a specific severity level. Several factors contribute to this inconsistency in reporting intensity perception and also shed light on why parameters other than knee momentum and A-P CoP did not show an association with users’ subjective perception. Here are some key points to consider:

1. The introduced disturbance is only 200ms, and users’ quick responses can mitigate some of the effects of disturbance which can affect gait stability measurements and their own perception. For instance, the movement of the contra-lateral leg during the swing phase dominates the measurement of the full-body angular momentum [47]. A quick hip recovery motion from the contra-lateral side can reduce the effect of the generated disturbance on full-body angular momentum. So, these recovery efforts may lead to mitigating the effect of the disturbance on some gait stability metrics and making them less sensitive to the disturbance.
2. Furthermore, the previous study [35] found that the introduced disturbances were novel experiences for participants and they kept changing their strategies to recover from the disturbances. This inconsistency in recovery efforts can lead to large variations in the measured stability metrics and participants’ own perceptions. Eventually, these large variations make it harder to identify the association between objective metrics and subjective perceptions.
3. Some gait stability metrics incorporate contributions from multiple body segments, which can reduce their sensitivity to joint-level disturbances. On the other hand, local measurements such as knee momentum are more sensitive to joint-level disturbances because they directly capture the dynamics and interactions at the disturbed joint. The A-P CoP only focused on the prosthetic side CoP; so, it can be considered as a local measurement.

One limitation of this study is the relatively small sample size of participants and it is possible that a larger sample size could provide more comprehensive results. Furthermore, the study only evaluated metrics during level-ground walking and did not consider other walking conditions, such as inclines or uneven terrain.

## V. FUTURE DIRECTION

The findings of this study may have implications for the design of the controller of robotic assistive devices, particularly in terms of addressing internal disturbances that may result in harmful consequences. In this regard, accurate and reliable gait stability indicators can be employed to identify appropriate interventions in the event of device malfunctions in order to enhance individuals’ gait stability. This study suggests selecting a metric that is associated with the human perception of gait stability. Then, the internal disturbances can be compensated/mitigated to the level that affects the user’s gait. Some factors are needed to consider in choosing a metric for control purposes, such as cost and difficulty of measuring using a wearable system. Also, for real time applications, the choice of filter parameters should be made in consideration of the desired trade-off between noise reduction and minimal delay, depending on the specific application requirements.

Future studies could examine the generalizability of the findings across different robotic assistive devices, as well as explore the potential transferability to other forms of disturbances.

Finally, the current study highlights the importance of user-centered design in the development of robotic assistive devices. By prioritizing the preferences of individuals with gait stability/balance impairments, designers can create devices that effectively enhance users’ safety and independence.

## Supplementary Material

supp1-3328877

## Figures and Tables

**TABLE I:** Demographic Information of Individuals With Transfemoral Amputation Involved in the Study

Subjects	Age (Yrs)	Weight (Kg)	Height (m)	Affected Side	Reason of Amputation	k-Level
TF01	24	86	1.67	Left	Congenital	K4
TF02	43	66	1.73	Left	Trauma	K4
TF03	71	66	1.65	Left	Trauma	K3 or K4
TF04	45	54	1.64	Left	Trauma	K3
TF05	23	75	1.80	Left	Congenital	K4
TF06	40	82	1.82	Left	Trauma	K4
TF07	49	72	1.73	Left	Trauma	K3 or K4

**TABLE II:** The Number of Times Each Subjects Reports the Impact of Each Disturbance Condition on Their Gait Stability During the Experiments

Subjects	Perception			
	None	Small	Medium	Large
**TF01**	42	44	34	42
**TF02**	46	51	45	20
**TF03**	46	44	50	30
**TF04**	36	38	51	43
**TF05**	14	48	69	13
**TF06**	13	73	58	12
**TF07**	8	63	43	42
	29 ± 17	52 ± 12	51 ± 12	29 ± 14
