# Supplementary material for: Investigating the Association of Quantitative Gait Stability Metrics With User Perception of Gait Interruption Due to Control Faults During Human-Prosthesis Interaction
Source: IEEE Trans Neural Syst Rehabil Eng. Author manuscript; Available in PMC 2023 Dec 25. (PMC10749666; doi:10.1109/TNSRE.2023.3328877)
Supplement: supp1-3328877 [file NIHMS1949392-supplement-supp1-3328877.pdf]

TABLE III: Kendall correlation coefficients assessing the association between gait stability metrics and users' perception when disturbance was applied as flexion type.

**Subjects**	**Kendall Correlation Coefficient**							
	**Step Length**	**Step Width**	**A-P CoP**	**Inclination Ang**	**Stability Margin**	**Knee Momentum**	**Body Momentum**	**V-CoM**
**TF01**	-0.04	-0.17	0.35**	0.22*	0.14	0.38***	0.34***	0.15
**TF02**	0.07	0.04	0.28*	-0.04	0.10	0.44***	0.05	0.13
**TF03**	-0.10	0.11	0.24*	-0.05	0.03	0.06	0.13	0.05
**TF04**	-0.09	0.06	0.03	-0.26*	-0.02	-0.10	-0.06	-0.14
**TF05**	0.01	0.13	0.16	0.10	0.26*	0.00	0.17	0.11
**TF06**	0.16	0.14	-0.14	-0.15	-0.31**	0.27*	0.05	-0.08
**TF07**	-0.01	0.03	0.49***	0.35**	0.48***	0.55***	0.40***	0.55***

TABLE IV: Pseudo-R Squared result of the generalized linear model fit for gait stability metrics and users' perception when disturbance was applied as flexion type.

**Subjects**	**Pseudo-R squared of logistic regression models (R^2_{GLM}) for flexion type disturbance**							
	**Step Length**	**Step Width**	**A-P CoP**	**Inclination Ang**	**Stability Margin**	**Knee Momentum**	**Body Momentum**	**V-CoM**
**TF01**	0.04	0.02	0.22	0.04	0.04	0.24	0.11	0.06
**TF02**	0.02	0.03	0.29	0.03	0.00	0.27	0.01	0.01
**TF03**	0.00	0.01	0.27	0.03	0.04	0.04	0.05	0.00
**TF04**	0.01	0.01	0.05	0.05	0.03	0.05	0.02	0.02
**TF05**	0.01	0.02	0.09	0.04	0.04	0.13	0.03	0.02
**TF06**	0.03	0.03	0.02	0.02	0.11	0.22	0.03	0.04
**TF07**	0.03	0.07	0.22	0.12	0.17	0.33	0.14	0.30

TABLE V: Kendall correlation coefficients assessing the association between gait stability metrics and users' perception when disturbance was applied as extension type.

**Subjects**	**Kendall Correlation Coefficient**							
	**Step Length**	**Step Width**	**A-P CoP**	**Inclination Ang**	**Stability Margin**	**Knee Momentum**	**Body Momentum**	**V-CoM**
**TF01**	0.03	0.07	0.44***	0.015	-0.12	0.56***	-0.14	0.13
**TF02**	0.07	-0.07	0.41***	0.06	0.08	0.19	0.19	0.05
**TF03**	-0.06	0.04	0.18	-0.16	0.16	0.49***	0.21	0.30**
**TF04**	0.18	0.18	0.02	0.19	0.08	0.51***	0.32***	0.24*
**TF05**	-0.07	0.18	-0.01	0.14	0.14	0.50***	-0.01	0.08
**TF06**	0.15	-0.04	0.49***	0.29**	-0.10	0.02	0.14	-0.27*
**TF07**	0.06	0.019	-0.09	-0.08	-0.07	-0.10	0.05	-0.28*

TABLE VI: Pseudo-R Squared result of the generalized linear model fit for gait stability metrics and users' perception when disturbance was applied as extension type.

**Subjects**	**Pseudo-R squared of logistic regression models (R^2_{GLM}) for extension type disturbance**							
	**Step Length**	**Step Width**	**A-P CoP**	**Inclination Ang**	**Stability Margin**	**Knee Momentum**	**Body Momentum**	**V-CoM**
**TF01**	0.00	0.01	0.29	0.00	0.02	0.37	0.01	0.03
**TF02**	0.00	0.01	0.28	0.01	0.02	0.04	0.03	0.01
**TF03**	0.08	0.03	0.06	0.00	0.00	0.24	0.05	0.01
**TF04**	0.02	0.00	0.00	0.03	0.07	0.23	0.11	0.05
**TF05**	0.04	0.03	0.00	0.01	0.05	0.25	0.06	0.02
**TF06**	0.01	0.05	0.24	0.07	0.13	0.04	0.02	0.13
**TF07**	0.00	0.00	0.03	0.02	0.02	0.01	0.04	0.02
